# Interaction between Insects, Toxins, and Bacteria: Have We Been Wrong So Far?

**DOI:** 10.3390/toxins10070281

**Published:** 2018-07-06

**Authors:** Guillaume Tetreau

**Affiliations:** 1 University of Perpignan, IHPE UMR 5244, CNRS, IFREMER, University of Montpellier, F-66860 Perpignan, France; guillaume.tetreau@gmail.com; Tel.: +33-045-742-8634; 2 University Grenoble Alpes, CNRS, CEA, IBS, F-38000 Grenoble, France

**Keywords:** invertebrate immunity, host-pathogens interaction, toxins, *Bacillus thuringiensis*, resistance

## Abstract

Toxins are a major virulence factor produced by many pathogenic bacteria. In vertebrates, the response of hosts to the bacteria is inseparable from the response to the toxins, allowing a comprehensive understanding of this tripartite host-pathogen-toxin interaction. However, in invertebrates, this interaction has been investigated by two complementary but historically distinct fields of research: toxinology and immunology. In this article, I highlight how such dichotomy between these two fields led to a biased, or even erroneous view of the ecology and evolution of the interaction between insects, toxins, and bacteria. I focus on the reason behind such a dichotomy, on how to bridge the fields together, and on confounding effects that could bias the outcome of the experiments. Finally, I raise four questions at the border of the two fields on the cross-effects between toxins, bacteria, and spores that have been largely underexplored to promote a more comprehensive view of this interaction.

## 1. Introduction

Many pathogenic bacteria produce toxins that are generally considered their principal virulence factor [1,2]. They help to circumvent the host immune system and to promote bacterial niche establishment by disrupting host cell tissues and/or by controlling their competitors’ population [2,3,4]. Therefore, they directly participate in bacterial replication and transmission to new hosts [3]. Not all toxinogenic bacteria have received equal interest. In vertebrates, the most studied are those responsible for deleterious human diseases while in invertebrates, research is focused on the most virulent ones usable as biological insecticides for pest control [5]. This led to a restricted list of pathogens to become biological models for a wide range of experiments spanning different fields of research.

Toxinogenic bacteria targeting vertebrates, especially humans, have received reasonably special attention [2]. Toxins are generally produced during host infection and most of them have been known to interact with the immune system, either directly because immune cells are their primary target, or indirectly as the immune system reacts to toxin activity on the host’s tissues [6]. Therefore, in vertebrates, the study of the mechanisms of response to the toxin is inseparable from the immune response to the pathogen. Such integrative approach allows deciphering the fine regulatory mechanisms underlying the evolution of host-pathogen interaction. In invertebrates, however, the adaptation to toxinogenic bacteria has been investigated by two complementary but distinct fields of research: toxinology and immunology. While toxinologists consider that toxins are the major toxic component that drives the adaptation of the insect host, mostly neglecting the role of the bacteria, immunologists rather deem that toxins are just one virulence factor among others and that the real adaptation of the insects is to the bacteria through its immune system.

**Figure 1:**
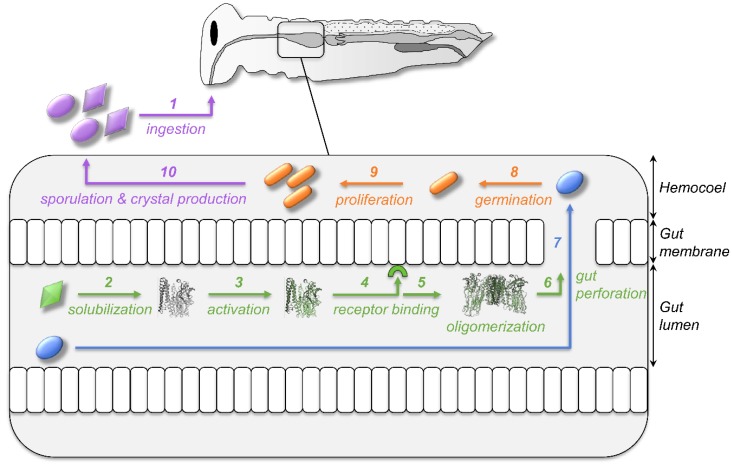
From ingestion to infection—the multistep mode of action of *Bacillus thuringiensis*. *Bt* is ingested by the insect as a suspension of bacterial spores and crystals of toxins (purple, step 1). Crystals (green) are solubilized into the alkaline gut of insects (step 2), leading to the release of protoxins that are activated by gut enzymes into toxins (step 3). They will then bind to specific receptors at the surface of the gut cell membrane (step 4), allowing toxin oligomerization (step 5) and pore formation leading to gut perforation (step 6). Spores (blue) can then reach the hemolymph by going through the damaged gut (step 7). They can germinate (step 8) and the bacteria (orange) proliferate (step 9) to infect the whole insect body. The decrease in the food source will trigger the sporulation of the bacteria and the simultaneous production of crystals of toxins (step 10). Spores and crystals will be eaten by a new host for the life cycle of the *Bt* to continue.

Among the toxinogenic bacteria targeting insects, the spore-forming bacterium *Bacillus thuringiensis* (*Bt*) is by far the most studied, notably because it is the most used biocontrol agent for agricultural pests and disease-carrying insects [7]. *Bt* is genetically indistinguishable from the two human pathogens *B. cereus* and *B. anthracis* (forming the *B. cereus* group) and it only differs by the production during its sporulation of a crystal of invertebrate-specific toxins, whose genes are located in plasmids [8,9]. *Bt* became an unavoidable model of gram-positive bacteria due to the easiness to maintain and grow *Bt* using artificial media in the lab and to produce and store its toxins as crystals, despite its very specific sequential multi-step mode of action (Figure 1). *Bt* produces pore-forming toxins (PFTs) that disrupt the host’s gut (steps 1 to 6) to trigger the colonization of the hemolymph by the spores (step 7) in which they can germinate and bacteria proliferate (steps 8 to 9) in order to produce new crystals during sporulation (step 10) [10,11]. The complexity and specificity of *Bt* ecology and mode of infection, notably its capacity to make spores and the fact that the fitness cost of toxin production is born by the parental generation [12], require a lot of caution when using *Bt* as a model for immunological and toxinological experiments. Observations made on host response to *Bt* might not be universal and readily expandable to all gram-positive bacteria or even all bacteria, as it is often done. Only considering a part of the host response, solely to the toxins, to the bacteria, or to the spores, might, therefore, lead to partial and/or erroneous conclusions.

By using *Bt* as a case study, I will highlight how such a dichotomy between toxinology and immunology led to a biased view of the ecology and evolution of the interaction between insects and toxinogenic bacteria. I will present the historical reason behind such a dichotomy and the difficulties encountered to reconcile the two fields, from different semantics to potentially confounding effects generally omitted that can bias the outcome of experiments. In the light of recent advances in each field, I will propose future scientific directions yet largely unexplored to bridge together these two fields to provide a comprehensive and integrative view of this interaction.

## 2. A Historical Dichotomy

**Figure 2:**
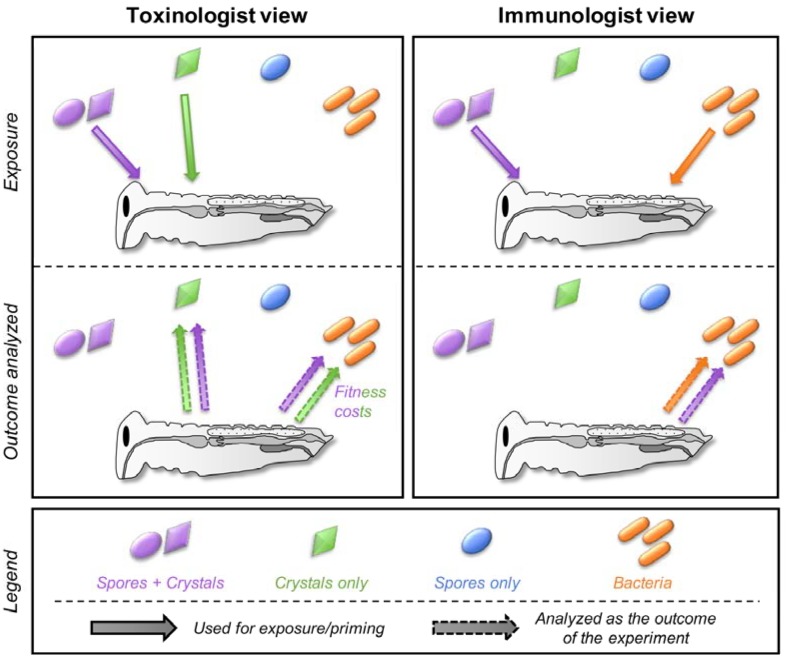
The toxinologist and immunologist views of the interaction between insects and toxins, spores and bacteria. This figure is a support for the different sections of the manuscript. The different views are discussed in Section 2. Spore + crystals, crystals only, spores only, and bacteria are indicated in purple, green, blue, and orange, respectively. The condition used for the exposure of the insect is indicated by a solid arrow from the spore/toxin/bacteria to the insect. This indicates whether the bacteria, spores, toxins, or a mixture of spores and toxins are generally used in each field (that is, the mixture spores + crystals and crystals of toxins alone for toxinologists while it is rather a mixture of spores + crystals and bacteria alone for immunologists). The outcome of the experiment measured is indicated by a dashed arrow from the insect to the spore/toxin/bacteria. This indicates which form of *Bt* has been studied and used as a proxy for the experiment. This is mainly the toxins for toxinologists and bacteria (immune fitness cost) while immunologists mainly focus on the bacteria and/or the immune response induced.

Historically, toxinologists and immunologists ended up studying *Bt* for different reasons. As a widely used biological insecticide, understanding the mechanism of action of the pore-forming toxins opens ways to improve its efficacy and has been the focus of many toxinologists [13,14]. In contrast, with this applied research objective, immunologists found in *Bt* a convenient and ubiquitous model of a gram-positive bacterium for controlled infection experiments to investigate invertebrate immunity. In each field, experiments were generally designed with only one of the two factors (toxins or bacteria) in mind and the results were analyzed through the very same filter (Figure 2).

In both fields, a mixture of spores and crystals was generally used for insect exposure but the parameters measured in insects differed depending on the field (Figure 2). They investigated either the immune response (survival to reinfection with bacteria, antimicrobial activity, expression of immune genes, prophenoloxidase activity, and so forth) or the evolution of toxin resistance (bioassays with the toxin(s), expression of gut membrane receptors, gut enzymes activity, and so forth). Obviously, measuring all these parameters in a single study can be laborious and might be out of the scope of the study. Nevertheless, a too simplified interpretation of the results is often visible in many studies (especially in the title and abstract) from both fields, including, I must admit, even in some articles that I authored. This can be misleading by restricting the conclusions to the expected outcome (for example, by claiming that a toxin effect is observed when a mix of spore+crystal is used for exposure without investigating a potential effect on bacteria and vice versa). Authors must be careful during the experimental design to include proper controls and to use the factors adapted to the question (spore, toxins, bacteria, spore+toxins) in order to limit confounding effects that might bias the outcome of the experiment and lead to misinterpretation due to untested parameters.

## 3. When Semantic Misleads More Than It Informs

**Figure 3:**
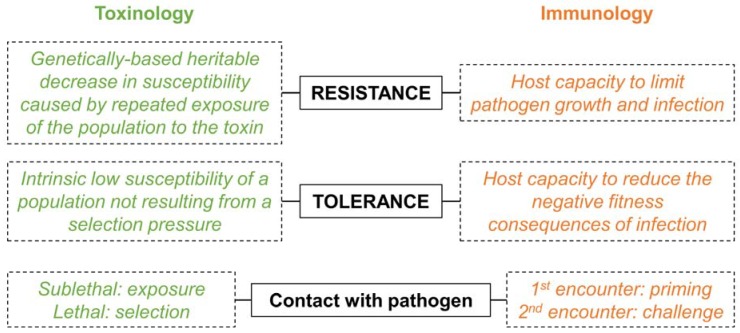
The key definitions that differ between the two fields.

The very first (and major) barrier when it comes to unifying different fields is a straightforward and universal semantic. The use of the same word with different meanings can be misleading and cause misinterpretations, especially when authors and readers are from different fields of research. The most notable example is the use of the words “resistance” and “tolerance” that differs between toxinologists and immunologists (Figure 3).

For toxinologists, the difference between these two terms is based on the presence (resistance) or absence (tolerance) of a selection pressure that led to the increased insect survival observed in the presence of the pathogen [15,16]. In this case, tolerance is also sometimes referred to as “natural resistance” [16]. This is mostly because toxinologists consider *Bt* as a biological insecticide and, therefore, they embraced the nomenclature associated with insecticides and pest control [7]. For immunologists, it rather depends on whether the host directly fights the infection (resistance) or attenuates the fitness consequences of the infection (tolerance) [17,18]. The definition given by toxinologists is therefore populational and trans-generational while the one from immunologists is more focused on the mechanism of response to infection within individuals. While it would be impossible to change such a fundamental semantic from both fields, authors should consider explaining what they really mean by “resistance” and “tolerance” in their study. They should also define any additional terms that might be misleading for the reader to facilitate linking together the results obtained in the studies from different fields.

## 4. A Prerequisite: Disentangle Confounding Effects

During the last few years, research on toxins and invertebrate immunity made enormous progress. Some of these discoveries shed light on previously unsuspected potential confounding effects between long studied mechanisms and newly identified ones. For each field, I selected what I consider as the major potential confounding effect that can be observed. Identifying and characterizing these effects in each field is required before investigating more challenging questions at the border of the two fields.

### 4.1. Trans-Generational Immune Priming (TGIP) Versus Evolved Bacterial Resistance

The ability of parents to transfer their immunological experience to their offspring has been known since the 1800s in vertebrates [19]. It is mostly due to the transfer of maternal antibodies through the placenta, milk, or egg yolk, allowing for the protection of the offspring against infection from a few weeks to several months [20]. For a long time, invertebrates were thought to be devoid of such phenomenon, notably due to the absence of a vertebrate-like adaptive immune system (that is, the production of antibodies through the clonal division of specialized cells). Almost two decades ago, the first evidence of the transmission of immunity from invertebrate parents to their offspring was published [21]. Since then, this phenomenon called TGIP has been identified in a wide range of insect species in response to a large variety of pathogens [22]. This has deep implications in the study of the evolution of bacterial resistance in invertebrates. This means that when studying an evolved resistance to a pathogen after several generations of selection, the phenotype observed might be a combination of both TGIP and the selected mechanisms that could lead to a biased analysis of the results [5]. Moreover, it seems that TGIP does not exist in all insect species and, within the same species, that it depends on the developmental stage and on the pathogen used for priming [22]. Therefore, before studying an evolved resistance to a pathogen (and potential cross-resistance to the toxins), it is mandatory to characterize and quantify the capacity of the insect to transfer its immune experience to its offspring after sublethal exposure at different developmental stages to identify the part of TGIP in the evolved resistance phenotype analyzed.

To date, the mechanisms by which insects achieve TGIP are still largely unknown. The direct transfer of antimicrobial peptides (AMPs) seems to play a major role in the immediate protection of the eggs [23], but long-term protection appears to require the transfer of “signals” stimulating the offspring’s own immune system [24]. The nature of these triggering signals is still under debate, specifically whether they are epigenetic modifications [25] or the direct transfer of bacterial peptides from the mother’s gut to the eggs [26]. Therefore, unraveling the molecular basis of TGIP in the insect species used would *i.* provide key data to the scarce knowledge on TGIP mechanisms and *ii*. unravel potential antagonistic/synergistic effects between TGIP mechanisms and selected ones, which has never been investigated to date.

### 4.2. One-Toxin Versus Multi-Toxins Resistance

Pest control using toxins has long relied on the dogma that the use of different toxins with contrasting modes of action induces an additive/synergistic selective pressure that will prevent/delay resistance development to both toxins [27,28]. This rationale is at the basis of the development of recent generations of genetically modified plants producing two or more toxins, called pyramids *Bt* crops [29]. Nevertheless, an increasing number of studies reported cases of cross-resistance between different toxins, with insects being able to survive multi-toxin exposure [30,31,32]. Selection with a mixture of toxins seems to induce mechanisms that none of the selections with toxins individually were inducing [33,34]. In addition, the reduced fitness associated with the presence of multiple toxins is not the simple addition of fitness reduction observed when exposed to toxins individually [35]. Altogether, these results indicate that assumptions based on the knowledge of each toxin individually do not perfectly match the observed phenotype of multi-toxin resistance. It is therefore important to investigate the effect of each toxin to be tested and of the different combinations of these toxins on the evolution of toxin resistance before investigating their potential cross-effects on the bacteria.

## 5. Cross-Effects between Toxins, Bacteria, and Spores

**Figure 4:**
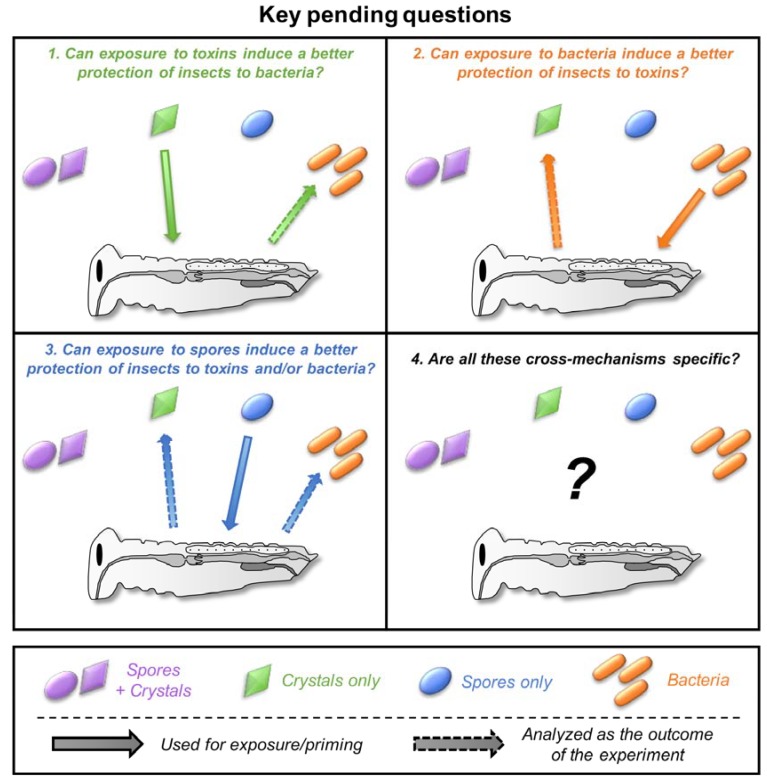
The key pending questions at the border of the two fields. Each number in the figure corresponds to the subsection numbers in Section 5. Spore + crystals, crystals only, spores only, and bacteria are indicated in purple, green, blue, and orange, respectively. The pictogram and color codes are the same as for Figure 2.

Each field extensively studied the details of insect-toxinogenic bacteria interaction using their own “codes”. Within each field, different methodologies, ways of designing, and ways of analyzing the experiments have been developed and accepted as the standards, which can differ between fields. This trend is driven by the system of peer-reviewing itself, as experts will review articles from their own field, influencing what is acceptable and considered as a standard while the very same approach could be considered as insufficient and/or out-of-scope for scientists from another field. Consequently, several trivial questions remain poorly explored when it comes to investigating the problematics at the border of the two fields (Figure 4). I listed four major questions below that I believe are the most promising and challenging for which some experiments already provide some support. This list is obviously subjective and hopefully expandable.

### 5.1. Can Toxins Trigger an Immune Response in the Host and Lead to the Protection against Bacteria?

For toxinologists, a modified immunity in resistant insects is generally perceived as a fitness cost (Figure 2). Indeed, selection experiments performed with spore/crystal mixtures or with toxins only that led to a high level of toxin resistance were generally associated with a constitutive depressed immunity in resistant insect strains [36,37,38]. In contrast, exposure of insects to low doses of *Bt* has been shown to induce the expression of different immune genes, including antimicrobial peptides [33,39,40,41,42], and to protect the insects from reinfection from different pathogens [43,44]. This suggests that toxins could induce an immune priming in the exposed insect and, therefore, affect the response to the bacteria during a challenge. This toxin-induced increased protection against pathogens can even be transmissible to the next generations [43,44,45] and could, therefore, be assimilated to TGIP [46]. Two possible mechanisms, not mutually exclusive, could explain this increased immunity upon toxin exposure:

i. Toxins, by disrupting the gut cells, release damage-associated molecular patterns (DAMPs) that can promote an immune response to infection [18,47,48]. They can be extracellular matrix components, such as peptidoglycan or glycoprotein, or intracellular molecules released from the cytosol, nucleus, plasma membrane, or organelles [49]. Among them, collagen and actin, which are cell-matrix junction proteins and cytoskeleton-extracellular matrix proteins, respectively, have been shown to be DAMPs in insects [50,51]. Meanwhile, they can also directly sense the presence of specific pathogens and trigger an immune response [52,53]. This dual role of DAMPs deserves deeper investigation in the context of the insect-toxin-bacteria interaction.

ii. Alternatively, toxins themselves could be directly sensed as “danger molecules” by the innate immune system of the host [54,55]. *Bt* has a sequential mode of action, requiring first the action of toxins in the midgut, then the development of bacteria in the hemolymph (Figure 1). Moreover, *Bt* spores always come with toxins in natura. As part of their co-evolution with *Bt* [56], it is sound that the insects might be able to directly sense the presence of the toxin itself as a warning for a bacterial infection to promptly occur in the hemolymph, providing crucial minutes for the insect to mount an appropriate immune response and increase its survival. Such direct immune recognition of toxins exists in vertebrates. Bacterial toxins can be recognized by canonical inflammasomes, such as *B. anthracis*’ toxins by the NLR family pyrin domain-containing proteins, triggering an immune response against both the toxins and the bacteria [57,58]. This hypothesis could be tested by engineering *Bt* toxins to impede pore formation while maintaining their overall tridimensional structure to verify if immune priming by toxins still occurs against bacteria when the “danger” is dissociated from the damages.

Several studies investigated insect immune response against *Bt* by directly injecting in the hemocoel cavity the bacteria, which are generally killed by heat or by formaldehyde treatment. This is supposed to mimic the bacterial antigen overload in the hemolymph characteristic of the last step of the bacterial infection process (Figure 2). By bypassing the gut disruption by toxins and their detection by larvae, a part of the priming can be missing and the resulting phenotype might not reflect the full potential of the insect to respond to infection [59].

### 5.2. Is the Immune System Involved in the Response to Toxins?

Some studies suggested that immune processes are at play for directly inactivating the toxins within the gut, involving toxin melanization and/or binding to lipophorins [41,45,60]. So far, it is unknown whether this phenomenon is common and shared by different insects or restricted to specific insect-pathogen interactions. Indeed, while melanization was clearly involved in toxin coagulation and was maternally transmissible in *Helicoverpa armigera* (Lepidoptera, Noctuidae) [45], it was rather a consequence of the increased immunity with little direct effect on the toxins in *Ephestia kuehniella* (Lepidoptera, Pyralidae) [60]. Interestingly, antimicrobial peptides such as defensins, which can be induced upon insect exposure to toxins [33,39], have recently been shown to directly inactivate bacterial toxins in humans [61,62]. In vertebrates, toxins can be targeted by both the innate and the adaptive immune system, as it is the case against *Clostridium difficile* toxins [63,64].

Altogether, the discrepancy between the increased immune response when insects are exposed to low doses of toxins and the constitutive decreased immunity observed in toxin-resistant selected insect lines suggests that the toxin-induced role of the immune system is rather inducible, potentially acting against both the bacteria and the toxins through a vertically transmissible mechanism yet to be more deeply and widely investigated.

### 5.3. Do Spores Play an Unsuspected Major Role in the Response to Bt Toxins and Bacteria?

Spores are highly resistant, non-reproductive forms of bacteria that are produced when the food source is becoming limited [65]. In most experiments, they are used in concert with crystals of toxins for exposure of insects but they are generally considered dormant forms and, therefore, their active role in the host-pathogen interaction received little attention. Yet, there are three main complementary reasons explaining why and how they could play a non-negligible role in the host response to bacteria and toxins:

i. Gut and hemocoel represent two distinct compartments within the insect. Even if they both have their own immunity, relying on different mechanisms triggered by different signals, they are finely interconnected. Indeed, ingestion of a pathogen unable to cross the gut barrier can trigger an elevated immune response in the hemocoel [66]. Therefore, the spore-induced gut immune response can trigger an immune response in the hemocoel to control bacterial infection.

ii. Although toxins alone can kill insects when provided in sufficient quantity, spores are known to synergize toxins even when they have been inactivated by γ-irradiation [67,68,69]. Interestingly, it has been evidenced that toxins can be incorporated on spore surface, providing the spore with antigens from the toxins [70,71,72,73]. Therefore, insects are exposed to toxin antigens even in the absence of the toxins themselves. Spores might be able to trigger specific toxin-inducible patterns and a trained immunity allowing an increased insect survival to toxins and bacteria.

iii. The simple presence of spores in the gut could induce physiological and metabolic modifications in the host that could affect its response to subsequent challenges with bacteria and/or toxins. A possible cause is that the ingestion of the pathogen induces a disturbance of the gut microbiota that is sensed by the host, which mounts an immune response in the gut and/or the hemocoel [74]. The role of the microbiota on *Bt* toxicity is still under debate [67,75,76]. However, a recent article revealed that the exposure to a mixture of spores and crystals induced a modification of microbiota composition in the surviving mosquito larvae but, again, the effect of spores and crystals could not be distinguished [77]. In human-flora-associated rats, oral exposure to spores of pathogenic *B. cereus*, but not of non-pathogenic *Bt*, also induced a modification of microbiota [78,79]. Interestingly, microbe-free insect larvae exhibited a lower survival upon reinfection with *Bt* spores than larvae with intact microbiota, supporting the role of microbiota in immune priming [80]. Further experiments with spores and toxins used separately for exposure are required to investigate the triggering factors of this modified microbiota and the consequences on host tolerance to *Bt*.

### 5.4. How Specific Are These Cross-effects between Toxins, Bacteria, and Spores?

Each *Bt* subspecies exhibits a specific set of one or more toxins encoded by plasmids that have been evolutionarily selected to allow for a high host specificity [7]. It has already been shown that different *Bt* subspecies do not trigger the same immune response in exposed insects [81,82]. Similarly, insects exhibit specific responses to toxins from a *Bt* subspecies that evolved with them (and are toxic for them) compared to toxins from other *Bt* subspecies. It is, therefore, possible that the cross-effects between toxins and bacteria described above also exhibit specificity to some extent, which has not been investigated to date. For example, exposure to a *Bt* toxin non-toxic for an insect might not trigger the same immune response, whether it is against bacteria from a toxic or non-toxic *Bt* subspecies.

## 6. Conclusions

We need to design and perform the experiments differently and analyze the results differently by keeping in mind, at each step of the process, that both immunity and toxin-response mechanisms can be induced and involved in the host response to toxinogenic bacteria. After having uncovered key mechanisms in each field, scientists must now work in concert to bridge together these two complementary fields to finally provide a comprehensive view of the interaction between insects, toxins, and bacteria.
